# Reduced Personal Space in Individuals with Autism Spectrum Disorder

**DOI:** 10.1371/journal.pone.0146306

**Published:** 2016-01-27

**Authors:** Kosuke Asada, Yoshikuni Tojo, Hiroo Osanai, Atsuko Saito, Toshikazu Hasegawa, Shinichiro Kumagaya

**Affiliations:** 1 Research Center for Advanced Science and Technology, The University of Tokyo, Tokyo, Japan; 2 College of Education, Ibaraki University, Ibaraki, Japan; 3 Musashino Higashi Center for Education and Research, Musashino Higashi Gakuen, Tokyo, Japan; 4 Department of Cognitive and Behavioral Science, The University of Tokyo, Tokyo, Japan; UNC Chapel Hill, UNITED STATES

## Abstract

Maintaining an appropriate distance from others is important for establishing effective communication and good interpersonal relations. Autism spectrum disorder (ASD) is a developmental disorder associated with social difficulties, and it is thus worth examining whether individuals with ASD maintain typical or atypical degrees of social distance. Any atypicality of social distancing may impact daily social interactions. We measured the preferred distances when individuals with ASD and typically developing (TD) individuals approached other people (a male experimenter) and objects (a coat rack with clothes) or when other people approached them. Individuals with ASD showed reduced interpersonal distances compared to TD individuals. The same tendency was found when participants judged their preferred distance from objects. In addition, when being approached by other people, both individuals with ASD and TD individuals maintained larger interpersonal distances when there was eye contact, compared to no eye contact. These results suggest that individuals with ASD have a relatively small personal space, and that this atypicality exists not only for persons but also for objects.

## Introduction

Humans have their own preferred personal space, an “interpersonal distance”. For example, on a train with several seats available, many individuals prefer to take a seat that is at some distance from other people. Indeed, the presence of others at a close distance can cause heightened tension [1], and thus we usually maintain a reasonable degree of distance from others. In addition, personal space functions to modulate social interaction, and a certain interpersonal distance can reflect the relationship between the persons [2,3]. Maintaining an appropriate interpersonal distance is important for establishing effective communication and good interpersonal relations.

Autism spectrum disorder (ASD) is a neurodevelopmental disorder characterized by difficulties with social interaction and communication [4]. ASD is well known to be associated with social deficits, such as difficulties with theory of mind skills [5] and emotion processing on the basis of cues from others’ eyes [6]. Many studies have assessed social perception and cognition in laboratory settings (e.g., [7,8]). However, relatively few studies of social behaviors have been conducted in more naturalistic settings, and this is important to the study of personal space regulation.

Differences and abnormalities of preferred interpersonal distance could produce communication problems between individuals. For example, when others closely approach an individual with an overly small personal space in order to initiate social interactions, the individual might not consider the approaching others as possible communicative partners, because they would remain outside the individual’s personal space. Moreover, he or she might often invade others’ personal space, which might elicit difficulties and make others feel uncomfortable. It is therefore worth examining whether individuals with ASD have atypical personal spaces because this could impact the flow of social encounters in individuals with ASD, and have significant implications for building a better social life.

A small number of previous studies of adult-child interactions have assessed personal space in ASD, revealing that children with ASD showed shorter interpersonal distances than typically developing (TD) children and children with other intellectual disabilities [9,10]. These studies included only 2 to 6 children in each group. In addition, the IQ of participants was not reported. Since children with intellectual disabilities tend to maintain shorter interpersonal distances [11], matching intellectual levels between ASD and TD groups is essential. Recently, several studies with larger samples have been conducted matching intellectual levels between the groups and/or using a more rigorous research method to measure personal space. Using the stop-distance technique [12,13], researchers have measured comfortable or uncomfortable distances from others standing in front of the person. However, there is a discrepancy among the results of the previous studies. Two studies of adults did not find atypical interpersonal distances in ASD [14,15]. This lack of atypicality may be the result of learning through experience, because previous studies in ASD have found improvements with age in social skills [16,17]. Another study of children examined comfortable interpersonal distance, and found larger interpersonal distances for an ASD group than for a TD group [18]. This result contradicts earlier findings for observations of adult-child interactions. Therefore, studies focused on young individuals matched on intellectual level and using different procedures (e.g., asking about “uncomfortable” distances) are needed to gain a reliable insight into this issue.

In addition, other issues still remain. The first issue concerns whether atypical personal space in ASD is found not only for other people but also for objects. For example, when a person is in a very small room with furniture, he or she needs more space and feels uncomfortable even if no one else were in the room. We have a tendency to maintain some distance even from non-human objects. Previous studies of ASD have focused on “interpersonal” distance, and there is a need to know whether individuals with ASD show atypical preferred distances from both humans and objects. If such atypicalities are not found for objects, we can assume that social properties of human beings (e.g., face, eyes, a sense of presence) modulate personal space regulation and that this might differ between individuals with ASD and TD individuals, where individuals with ASD exhibit reduced social priority [19]. The second issue concerns whether personal space modulation occurs as a function of others’ social cues in ASD. Previous studies have reported that TD individuals made eye contact less often when they were close together than at a distance [20,21]. Several studies revealed that eye contact does not facilitate social cognitive performance (e.g., person categorization, face memory) in individuals with ASD [22,23]. Eye contact is one of the key modulators of personal space, and it is important to know whether individuals with ASD are able to use this cue. The third issue concerns what factors relate to preferred interpersonal distance. One study found a positive correlation between preferred interpersonal distance and the degree of social anxiety in ASD [15]. More studies are needed that focus on other clinical complaints (e.g., social withdrawal) in addition to anxiety, to determine the clinical factors that affect preferred interpersonal distance.

The present study investigated the distances from i) other people (a male experimenter) and ii) objects (a coat rack with clothes), at which adolescents (ASD, TD; 12 to 19 years) felt uncomfortable due to proximity. We used the stop-distance technique, and the participants were asked to stop another’s approach or to stop walking when they felt uncomfortable. During the experiments, the experimenter either looked at the participants’ eyes or did not do so, in order to assess potential eye contact effects. In addition, we explored the relationships between preferred interpersonal distance and clinical complaints, such as anxiety.

## Method

### Ethics statement

Written informed consent was obtained from the participants and their parents. This study was approved by the Research Ethics committee of the University of Tokyo.

### Participants

Data from sixteen adolescents with ASD (15 males and 1 female) and 16 TD adolescents (15 males and 1 female) were analyzed (Table 1). The individuals with ASD had been diagnosed with autistic disorder (9), Asperger’s disorder (1), or pervasive developmental disorder (without a more specific diagnosis, 6) based on the DSM-IV criteria by at least one child psychiatrist, pediatrician, or psychologist [24].

**Table 1:** Participant characteristics.

	ASD		TD	
	Mean (SD)	Range	Mean (SD)	Range
**Age (years)**	15.7 (1.6)	13.5–18.2	14.4 (1.9)	12.4–19.4
**IQ**	100.1 (17.7)	75–130	105.8 (11.3)	82–118
**SCQ**	23.9 (8.0)	8–38	2.4 (2.2)	0–7
**ADOS**	12.5 (3.4)	7–18	–	–

ASD, autism spectrum disorder; TD, typically developing; SCQ, Social Communication Questionnaire; ADOS, Autism Diagnostic Observation Schedule. The ADOS scores were not obtained from the TD group.

The Autism Diagnostic Observation Schedule (ADOS, [25]) was administered to all the individuals with ASD. The Japanese version of the Social Communication Questionnaire (SCQ, [26]) was completed by the parents of all participants, to provide further assessment of autistic traits. The ADOS scores (Communication + Social Interaction Total) of all the individuals with ASD were within the ASD range (7 or greater), and the SCQ scores of all the TD individuals were within the normal range (less than 15). As expected, SCQ scores significantly differed between the groups (*F*(1, 30) = 107.845, *p* < .001, *η*_*p*_^2^ = .782; Table 1).

An abbreviated version of the Japanese Wechsler Intelligence Scale for Children (WISC-III) or Wechsler Adults Intelligence Scale (WAIS-R) was administered to all participants for measuring IQs [27,28]. All the individuals had IQs of 75 or higher. Except for the 32 individuals described above, an additional three TD individuals who had higher IQs than others in each sex group were excluded from the analysis in order to match sex ratio and mean IQ across the groups. Therefore, IQ did not significantly differ between the groups (*F*(1, 30) = 1.181, *p* = .286, *η*_*p*_^2^ = .038; Table 1).

In order to examine the relationship between preferred interpersonal distance and clinical complaints, such as anxiety, the parents completed the Child Behavior Checklist (CBCL, [29]) and responded to questions about their children’ current situation or the situation during the past 6 months. We only administered the Anxiety/Depressed (13 items), Withdrawn/Depressed (8 items), Somatic complaints (11 items), Social problems (11 items), and Attention problems (10 items) subscales of the CBCL, to make the process easier for parents. Parents’ responses to each item were scored as follows: 0 (*Not true*); 1 (*Somewhat or sometimes true*); or 2 (*Very true or often true*). The total score for each subscale was used in the data analysis.

### Procedure

All participants were tested individually. We measured the distance from the experimenter or an object at which participants felt uncomfortable due to proximity, as an index of the personal space. Distances from the participants’ toe to the experimenter’s toe or the object were measured using a digital laser measure (Bosch, GLM80) or a tape measure (only when the digital laser measure could not be used due to closeness over the controllable range). The experimenter initially stood 6 m away from the participants. As a practice trial, the experimenter walked to the participants while looking toward the participants’ eyes, and asked them to say stop when they felt uncomfortable.

After that, the participants completed four blocks of trials that comprised a 2 (Approacher: Experimenter/Participant) x 2 (Eye Contact: Eye Contact/No Eye Contact) design. In half of the trials, the experimenter walked to the participants, and in the other half of the trials, the participants walked to the experimenter. In addition, in half of the trials, the experimenter looked toward the participants’ eyes, and in the other half of the trials, the experimenter looked down. We also measured the preferred distance to an object, to examine whether characteristics of personal space were limited only to humans. In this block (object condition), participants walked to a coat rack with similar colored clothes to the ones worn by the experimenter. The experimenter asked the participants to say stop or stop walking when they felt uncomfortable due to the closeness to the examiner or object. If the participants did not say stop or stop walking, the preferred distance was 0 m. Such responses were only shown in the object condition by one individual with ASD (3 trials) and one TD individual (2 trials). When the experimenter walked to the participants, the experimenter tried to take steps of approximately 45 cm a second and walk at a constant speed for each experiment. When the participants walked to the experimenter or the object, they were not instructed on the speed at which they should walk so that they could walk naturally and concentrate on when they stopped. The participants completed the experimenter approaching condition, participant approaching condition, and object condition in a fixed order in order to increase the participants’ understanding of the procedure. Only the order of the eye contact conditions was randomized.

Another block was administered to a subset of the participants (ASD: 11, TD: 9) to confirm whether the same results were seen when the experimenter backed away from a closer position to the participants. In this block, the experimenter stood close to the participants (30 cm) and backed away while making eye contact. The experimenter tried to take steps of approximately 40 cm a second and back away at a constant speed for each experiment. After one practice trial, the participants were asked to say stop when the interpersonal distance became no longer uncomfortable. An additional one individual with ASD was excluded from the analysis because this participant did not say stop and therefore the preferred distance could not be measured.

All participants conducted the experiment in the same booth (a 7.9 x 3.7 m space separated by partitions) and worked with the same male experimenter, who wore the same clothes during all the experiments. None of the participants had previously met the experimenter. Each block consisted of three trials, and therefore each participant completed 15 or 18 trials in total.

## Results

**Fig 1:**
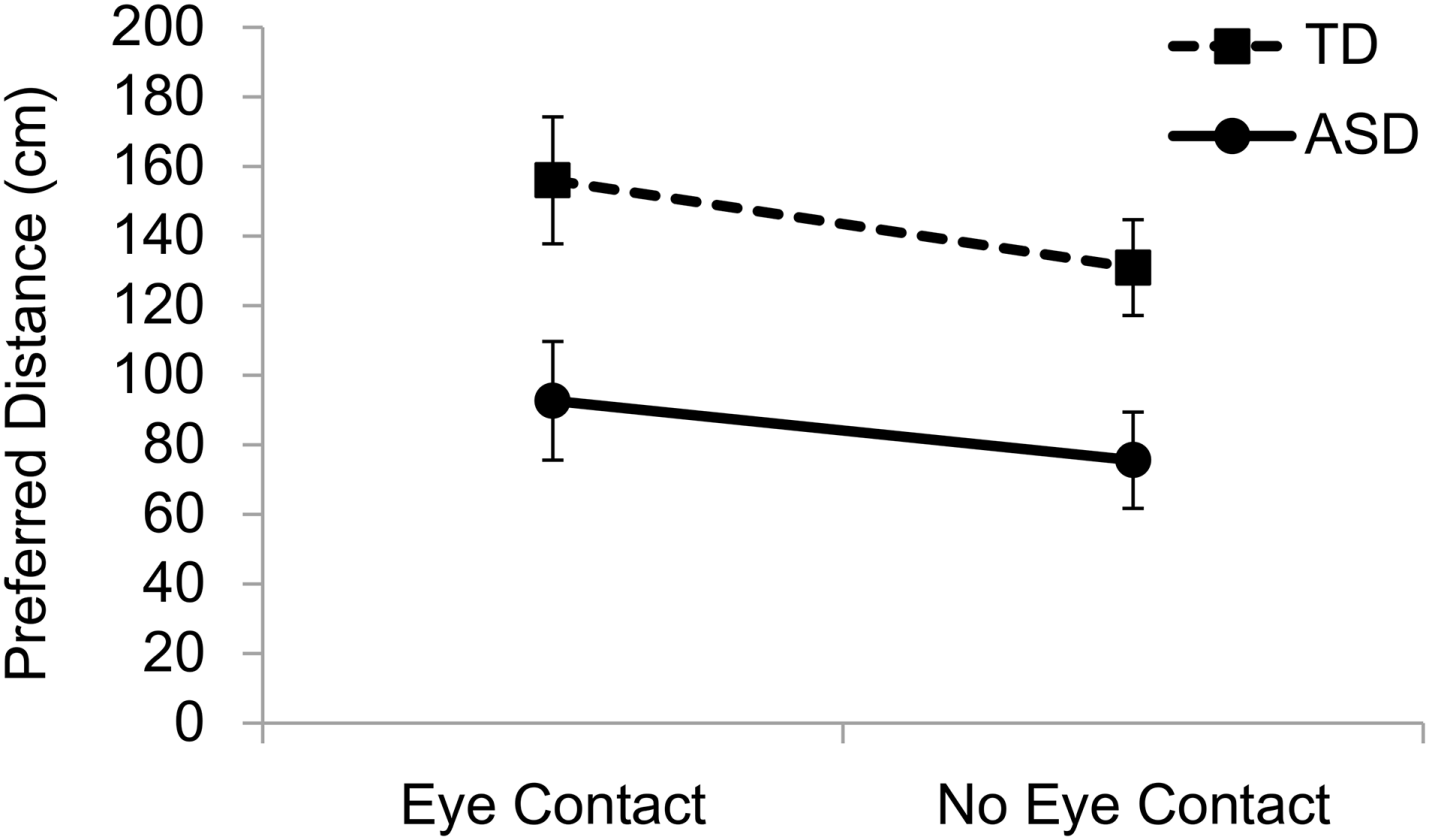
Mean preferred distances from another person when the other person approached. ASD, autism spectrum disorder; TD, typically developing. Error bars indicate standard errors of the mean.

**Fig 2:**
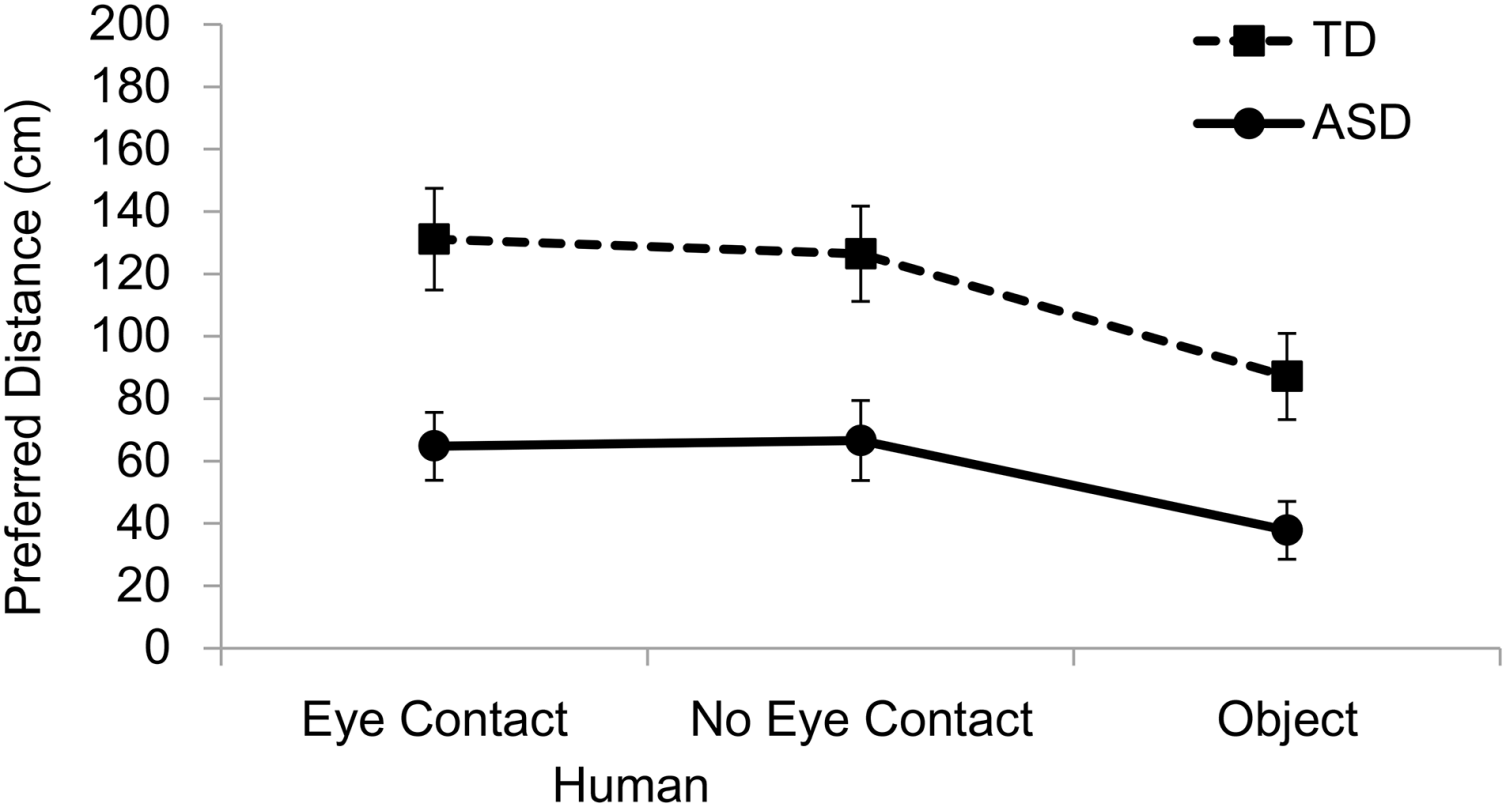
Mean preferred distances from another person and an object when the participants approached. ASD, autism spectrum disorder; TD, typically developing. Error bars indicate standard errors of the mean.

We used the mean of the three trials in each block for the analyses. To examine the effects of approacher and eye contact, a Group (ASD, TD) x Approacher (Experimenter, Participant) x Eye contact (Eye contact, No eye contact) three-way ANOVA was carried out for preferred distance (Figs 1 and 2). This analysis revealed a significant main effect of Group (*F*(1, 30) = 9.610, *p* = .004, *η*_*p*_^2^ = .243). Individuals with ASD showed significantly shorter interpersonal distances than TD individuals. This analysis also revealed significant main effects of Approacher (*F*(1, 30) = 14.473, *p* = .001, *η*_*p*_^2^ = .325) and Eye contact (*F*(1, 30) = 5.034, *p* = .032, *η*_*p*_^2^ = .144) as well as a significant Approacher x Eye contact interaction (*F*(1, 30) = 7.928, *p* = .009, *η*_*p*_^2^ = .209). Follow-up ANOVAs revealed that when the experimenter approached the participants, both groups showed significantly longer interpersonal distances in the eye contact condition than in the no eye contact condition (*F*(1, 30) = 7.606, *p* = .010, *η*_*p*_^2^ = .202). However, this significant difference was not seen when the participants approached the experimenter (*F*(1, 30) = .128, *p* = .723, *η*_*p*_^2^ = .004). There were no other significant main effects or interactions.

To examine the effect of the targets that the participants approached, we compared the distance to the other person without making eye contact and the object (Fig 2). A Group (ASD, TD) x Target (Participant approacher with no eye contact, Object) two-way ANOVA on preferred distance revealed a significant main effect of Group (*F*(1, 30) = 10.030, *p* = .004, *η*_*p*_^2^ = .251), showing shorter distances from the targets in the ASD group compared to the TD group. This analysis also revealed a significant main effect of Target (*F*(1, 30) = 28.765, *p* < .001, *η*_*p*_^2^ = .489), showing shorter distances from the object than from the experimenter in both groups. There was no significant interaction.

Interpersonal distance was also examined when the experimenter backed away from a close position for a portion of the participants (ASD: 11, TD: 9). We found the same trend, that is, shorter interpersonal distances in the ASD group than in the TD group (ASD: *M* = 83.6 cm (*SD*: 38.4), TD: *M* = 111.4 cm (*SD*: 52.8)). However, this difference did not reach significance (*F*(1, 18) = 1.854, *p* = .190, *η*_*p*_^2^ = .093).

Lastly, to examine whether age, IQ, autistic traits (SCQ and ADOS scores), and clinical complaints (CBCL scores) were related to interpersonal distance (mean for all human trials except for the experimenter backing away), we computed Pearson’s correlation coefficients (Table 2). One TD individual was excluded only from the analyses regarding CBCL scores because this individual was older than the target age range of the CBCL. Among all the analyses, the highest (negative) correlation was found between autistic traits (SCQ scores) and interpersonal distance (*r* = -.434, *p* = .013) for all participants. However, after Bonferroni’s correction for multiple analyses (*α* = .0063 (all participants), .0056 (ASD), and .0063 (TD), respectively), we did not find any significant correlations either for all participants or each group separately.

**Table 2:** Pearson’s correlation coefficients between interpersonal distance and age, IQ, autistic traits, and clinical complaints for all participants, the autism spectrum disorder group, and the typically developing group.

		**Age**	**IQ**	**SCQ**
**ALL**	**Correlation Coefficient**	-.130	-.095	-.434
	**p-value**	.477	.603	.013
**ASD**	**Correlation Coefficient**	-.205	-.101	-.044
	**p-value**	.445	.710	.871
**TD**	**Correlation Coefficient**	.267	-.419	.173
	**p-value**	.318	.106	.521
		**ADOS**	**Anxiety/Depressed (CBCL)**	**Withdrawn/Depressed (CBCL)**
**ALL**	**Correlation Coefficient**	–	-.061	-.087
	**p-value**	–	.744	.643
**ASD**	**Correlation Coefficient**	.260	.107	.229
	**p-value**	.330	.693	.394
**TD**	**Correlation Coefficient**	–	.204	-.015
	**p-value**	–	.466	.958
		**Somatic Complaints (CBCL)**	**Social Problems (CBCL)**	**Attention Problems (CBCL)**
**ALL**	**Correlation Coefficient**	-.063	-.218	-.028
	**p-value**	.736	.238	.880
**ASD**	**Correlation Coefficient**	.049	.050	.192
	**p-value**	.858	.854	.475
**TD**	**Correlation Coefficient**	.036	.305	.261
	**p-value**	.900	.269	.348

ALL, all participants; ASD, autism spectrum disorder; TD, typically developing; SCQ, Social Communication Questionnaire; ADOS, Autism Diagnostic Observation Schedule; CBCL, Child Behavior Checklist.

ALL: Numbers used for the analyses were 32 except for CBCL scores (31). ASD: Numbers used for the analyses were all 16. TD: Numbers used for the analyses were 16 except for CBCL scores (15). The ADOS scores were not obtained from the TD group.

## Discussion

The present study investigated personal space in individuals with ASD and those who were developing typically. Three main findings were obtained. First, individuals with ASD showed shorter preferred interpersonal distances than TD individuals. Second, the same tendency was found when the participants judged the preferred distance from an object. Third, both individuals with ASD and TD individuals preferred larger interpersonal distance when eye contact was established than when it was not, in the case of another person approaching them.

The results of reduced personal space in ASD is congruent with earlier observational studies of small samples [9,10], and may implicate the amygdala hypothesis of ASD [30,31]. This hypothesis posits that atypical amygdala structure and function affects social behaviors in ASD. A recent study found that the amygdala is involved in the regulation of personal space. Kennedy et al. [13] reported that an individual with bilateral amygdala damage showed strikingly short interpersonal distance. This individual did not feel uncomfortable even when nose-to-nose with the experimenter while making direct eye contact. Congruent evidence was reported from another line of ASD research using a social cognition task. In this study, Adolphs et al. [32] found that individuals with ASD rated unfamiliar faces as more approachable than TD individuals.

Our data suggest that atypically reduced personal space in ASD is not limited to other people. Individuals with ASD, compared to TD individuals, also showed shorter preferred distances from an object. This might indicate that individuals with ASD have difficulties with fear awareness in general, not only with social distance judgments. The amygdala is involved with both physical (e.g., avoiding snakes) and social (e.g., recognizing others’ emotion) fear awareness [33,34]. For example, an individual with bilateral amygdala damage experienced no fear of snakes and spiders [33] and had difficulties in recognizing fear in the facial expressions of others [34]. General deficits in fear awareness might be the basis for the atypical range of personal space observed in ASD. To know more about the neural underpinnings of personal space, we need more brain imaging or neurophysiological data. An fMRI study of TD individuals found greater amygdala activity when the experimenter was closer to the participants, compared to when further away [13]. Other fMRI studies for TD individuals revealed greater amygdala activation for social fears (i.e., responses to threatening faces) than non-social fears (i.e., responses to threatening scenes) [35,36]. In our study, both TD individuals and individuals with ASD maintained larger distances from another individual than from an inanimate object, and this might be related to different patterns of amygdala activation for social and non-social fears.

However, another interpretation of these results is possible. We conducted the experiments in a fixed order. As a result, there might have been a carry-over effect from the human conditions to the object condition, whereby the participants might have behaved as if there were another person in the object condition. The difference between the ASD and TD groups in the human conditions might have extended to the object condition. Therefore, caution is advised in applying the amygdala hypothesis to interpreting the results in the object condition, although participants might have experienced subtle fears also in the object condition because they bumped into the object if they did not stop walking.

Our data also suggest that individuals with ASD can use gaze cues during social interaction. In our study, when another person approached, individuals with ASD used eye contact information to judge what interpersonal distance they should take. In this situation, they might have been able to use eye contact cues because they did not need to respond rapidly and had enough time to process the information as the experimenter approached. Previous studies showed that if individuals with ASD did not need to respond rapidly or were explicitly instructed to look at others’ faces or eye region, some relatively typical social behaviors occurred (e.g., [37,38] for facial mimicry). The amygdala is also thought to be involved in the detection of eye contact [39,40]. The underlying mechanism of differential responses to eye contact shown by individuals with ASD needs to be further investigated. To gain a clearer insight about these issues, it is suggested that future ASD research should focus on the relationship between amygdala activation and distances that are maintained from humans and objects. In addition, possible implications of factors other than the amygdala, such as whether general distance estimation is impaired in ASD, should also be explored.

As for the relationships between personal space and clinical complaints, although Perry et al. [15] found a positive correlation between interpersonal distance and scores on a social anxiety questionnaire (the Leibowitz Social Anxiety Scale) in ASD, we did not find significant correlations between interpersonal distance and clinical complaints, including anxiety. We used a parent-report questionnaire (CBCL) and Perry et al. [15] used a self-report questionnaire. In addition, Perry et al. [15] used more items (24 items) for anxiety than we did (13 items), while we included other clinical complaints such as attention problems in addition to anxiety. Such differences might have affected the results.

As discussed above, our findings are consummate with earlier studies in ASD and the amygdala hypothesis. However, some recent results using the stop-distance technique were not congruent with our findings [14,15,18]. Studies of adults found greater variation in interpersonal distance in individuals with ASD than in TD individuals but no group mean difference [14,15]. However, in the same paper of Kennedy and Adolphs [14] using the stop-distance technique, they also used a questionnaire (the Social Responsiveness Scale) and reported that parents of children with ASD noted that their children tended to be too close to others and invade others’ personal space. Furthermore, Gessaroli et al. [18] examined children with ASD and showed the opposite result from ours; that is, larger interpersonal distances in individuals with ASD than in TD individuals.

IQ, autistic traits, age, and research methods could be potential factors affecting the regulation of personal space, and these might explain the discrepancy between the findings of the previous studies and the current study. IQ and autistic traits are unlikely to have caused the discrepancy, because the scores of these measures did not differ greatly between the studies (although Perry et al. [15] did not report them). In contrast, age might be an important factor. Previous studies suggest that individuals with ASD can improve their social skills through development [16,17]. The studies that did not find group differences were all of adult participants, whereas ours focused on the period of adolescence (12 to 19 years). In addition, research methods varied between the studies. The starting distance between the experimenter and the participants in the studies that were conducted with adult participants was almost half that used in our study. This difference might affect the results because the participants in those studies had less time to judge the social distance. Although the study of children by Gessaroli et al. [18] and the current study were done in very similar settings, the instructions to the participants were subtly different. In Gessaroli et al. [18]’s study, the participants were asked to respond at the most comfortable distance and larger interpersonal distances were shown by individuals with ASD. In ours, participants had to identify the distance at which they felt uncomfortable and shorter interpersonal distances were shown. Taken together, these studies suggest that younger individuals with ASD might be more tolerant with very close distances to others, while they may prefer a place that is at some distance from others. In order to clarify the discrepancy, more studies should be conducted on ASD using the stop-distance technique. Also, a meta-analysis of such studies may provide further clarity.

There are several limitations to this study. First, the experimenter was aware of the diagnostic information of the participants. Although care was taken to control the experimenter’s behavior by strictly following the experimental protocol, subtle differences in the experimenter’s behavior between the groups might have affected the results. An experimenter who is blind for diagnostic information would be preferable in future studies. Second, it is possible that shorter preferred distances shown by individuals with ASD stem from delayed responses. That is, if individuals with ASD responded more slowly in the experimenter approaching condition and stopped walking later in the participant approaching condition than TD individuals, it would produce the same results as in the current study. However, we believe that this was not the case. Although the small sample size in the experimenter backing away condition might have caused the group difference to be not significant, we found the same tendency even in this condition, in which delayed responses would have caused larger interpersonal distances.

Personal space differences between individuals with ASD and TD individuals leads us to pay attention to the daily social interactions of such people. The present study suggests that individuals with ASD might often invade the personal space of others, whereas they might not notice others in their socially proximal space or detect signals indicating that others intend to initiate a social interaction. Even if TD individuals approach individuals with ASD, individuals with ASD may fail to recognize that the approach invites communication. This might make TD individuals feel uncomfortable due to the invasion to their personal space, and individuals with ASD will miss opportunities to have social interactions. To know each other’s personal space is important for establishing effective communication and good interpersonal relations. It is suggested that further research should be conducted to explore this topic, by investigating individual differences that could improve theory and interventions related to personal space regulation and broader aspects of successful social functioning in ASD.

## Supporting Information

S1 Dataset The anonymized dataset used in the main analyses. (XLSX)
